# Seeing light at the end of the tunnel: Positive prospective mental imagery and optimism in depression

**DOI:** 10.1016/j.psychres.2016.11.025

**Published:** 2017-01

**Authors:** Julie L. Ji, Emily A. Holmes, Simon E. Blackwell

**Affiliations:** a Medical Research Council Cognition and Brain Sciences Unit, Cambridge, UK; b Department for Clinical Neuroscience, Karolinska Institutet, Sweden; c Mental Health Research and Treatment Center, Department of Psychology, Ruhr-Universität Bochum, Germany

**Keywords:** Mental imagery, Episodic future simulation, Optimism, Depression, Prospection

## Abstract

Optimism is associated with positive outcomes across many health domains, from cardiovascular disease to depression. However, we know little about cognitive processes underlying optimism in psychopathology. The present study tested whether the ability to vividly imagine positive events in one's future was associated with dispositional optimism in a sample of depressed adults. Cross-sectional and longitudinal analyses were conducted, using baseline (all participants, *N*=150) and follow-up data (participants in the control condition only, *N*=63) from a clinical trial (Blackwell et al., 2015). Vividness of positive prospective imagery, assessed on a laboratory-administered task at baseline, was significantly associated with both current optimism levels at baseline and future (seven months later) optimism levels, including when controlling for potential confounds. Even when depressed, those individuals able to envision a brighter future were more optimistic, and regained optimism more quickly over time, than those less able to do so at baseline. Strategies to increase the vividness of positive prospective imagery may aid development of mental health interventions to boost optimism.

## Highlights

• We examined the relationship between positive future imagery vividness and optimism.
• We analyzed cross-sectional and longitudinal data from a sample of depressed adults.
• Greater vividness of positive imagery was associated with greater current optimism at baseline.
• Greater vividness of positive imagery at baseline also predicted greater optimism seven months later.
• Positive mental imagery may be a useful target for optimism-focused interventions in mental health.

## 1 Introduction

A neglected area in psychopathology research concerns optimism and the ability to imagine a more positive future - something that may be of particular relevance to depression (Holmes et al., 2016). Most people are staunch “optimists”, expecting good rather than bad things to happen to themselves in the future (Weinstein, 1980). Defined as the generalized tendency to expect the future to turn out well, dispositional optimism is a robust predictor of psychological and physical wellbeing (Carver and Scheier, 2014, Carver et al., 2010). For example, people who are more optimistic are less likely to develop depressive symptoms (Giltay et al., 2006), and they recover from depression more quickly (Kronström et al., 2011). Given such findings, it is unsurprising that optimism has been the subject of extensive research – understanding its basis could not only illuminate important aspects of resilience, but also inform development of interventions to harness its beneficial effects. Numerous factors such as genetics (Bates, 2015, Plomin et al., 1992), life events (Broekhof et al., 2015), and socio-economic status (Heinonen et al., 2006) have been identified as contributing to whether an individual tends to be optimistic or otherwise. From a clinical perspective, cognitive processes involved in optimism may be particularly important to understand, as these could provide modifiable targets for psychological interventions.

Dispositional optimism is defined in terms of expectancies for the future, thus cognitive components of future-oriented thinking provide a useful focus for investigation. One important way in which people think about the future is via simulations of possible events using mental imagery (Schacter et al., 2008, Schacter et al., 2012). Mental imagery refers to internal representations of perceptual experience without external sensory input, commonly described as “seeing with the mind's eye”, “hearing with the mind's ear”, and so on (Kosslyn et al., 2001, Pearson et al., 2015). Theoretical accounts suggest that people may use the experience of simulating events, such as via mental imagery, as information to help evaluate and predict the future (e.g. Kahneman and Tversky, 1982; Szpunar and Schacter, 2013). Could differences in individuals’ expectancies about the future, that is, how optimistic or pessimistic they are, be related to how easily they can imagine different future possibilities? Depression is characterized by pessimistic expectancies for the future (e.g. Alloy and Ahrens, 1987; Beck et al., 1979; Miranda and Mennin, 2007), and emerging evidence suggests that people with depressed mood (Anderson and Evans, 2014, Holmes et al., 2008, Szőllősi et al., 2015) or major depressive disorder (Morina et al., 2011), struggle to generate images of positive events in their future. Specifically, they generate less vivid positive prospective mental images than non-depressed individuals. Conversely, initial research suggests that people who are optimistic can imagine positive events in their future particularly vividly (Blackwell et al., 2013).

Blackwell et al. (2013) examined the relationship between prospective mental imagery and optimism, using a cross-sectional design in a community sample (*N*=237). Higher levels of dispositional optimism, as measured via the Life Orientation Test – Revised (LOT-R; Scheier et al., 1994), were associated with higher subjective vividness of mental imagery for positive future scenarios (on the Prospective Imagery Task; PIT; Stöber, 2000), even when controlling for socio-demographic factors, health, general everyday mental imagery use, and vividness for negative future scenarios. This suggests the possibility that how vividly someone can imagine positive events in their future may be related to how optimistic they feel. This idea has intuitive appeal, to the extent that it may even appear tautological. However, the constructs measured are distinct: vividness as measured by the PIT is the perceived visual quality (clarity) of specific mental images generated, whereas dispositional optimism as measured by the LOT-R reflects the judgments one generally makes about one's own future, and makes no reference to imagery or imagined future events. A link between the quality of an individual's mental imagery and their generalized future expectancies is therefore interesting both from a theoretical perspective, contributing to our understanding of the role of imagery-based simulation in prospective thinking, and from a clinical perspective, highlighting a potential target for novel interventions. However, research in this area is preliminary, and many key questions remain.

The current study aimed to replicate the findings of Blackwell et al. (2013) and extend them in three main ways, using a sample of 150 depressed individuals from a clinical trial (Blackwell et al., 2015). First, we aimed to test whether the previously observed relationship between positive prospective imagery vividness and optimism also holds in a depressed sample. Baseline data from the trial (pre-intervention; *N*=150) allowed us to test the hypothesis that, amongst people with current major depression, higher vividness ratings for mental imagery of positive future scenarios (PIT) would be associated with higher optimism (LOT-R) when controlling for socio-demographic variables, health, general everyday mental imagery use, and negative imagery vividness. This provides a direct replication (Schmidt, 2009) of Blackwell et al. (2013), extended to a depressed sample.

Second, we aimed to rule out the possibility that the relationship between positive prospective imagery vividness and optimism would simply be a reflection of these measures’ shared variance with relevant, but previously unmeasured variables, such as depression, anxiety, or the negative cognitive biases with which these are associated. The trial data included measures of depression symptoms, trait anxiety, and interpretation bias, and thus we were able to test the hypothesis that a unique relationship between positive prospective imagery vividness and optimism would hold even when controlling for these additional related factors.

Third, we aimed to extend the previous cross-sectional research and probe *temporal* relationships between processes, a crucial step for addressing questions relating to mechanisms (Kraemer et al., 1997). The trial included longitudinal data, enabling us to investigate optimism over a seven-month period, using data from participants in the trial's control condition (*N*=63). We predicted that higher baseline positive prospective imagery vividness (PIT) would be associated with greater optimism seven months later (as indicated by higher scores on the LOT-R), even when controlling for baseline optimism scores and other relevant variables (cf. Kleim et al., 2014; Nelis et al., 2015).

## 2 Method

### 2.1 Participants

Participants were 150 depressed adults (103 female) recruited for a clinical trial (trial data reported in Blackwell et al., 2015; registered at clinicaltrials.gov, NCT01443234). Participants were recruited via advertisements in local media (newspapers and radio), web sites (e.g. Google, Facebook), and community (e.g. public library), university, and health settings (e.g. GP practices) in the local area. Advertisements had taglines such as “Feeling Blue? We need your help!” and included the information that the study involved completing an “online computer program” over a four-week period. People who responded to the advertisements by contacting the research team were emailed an information sheet about the study, and those interested in participating then completed screening questionnaires online. Potentially eligible participants (scoring 14 or above on the Beck Depression Inventory – II; BDI-II; Beck et al., 1996; i.e. the cut-off for mild depression) were invited for a face-to-face eligibility assessment. Five months into the trial, an additional brief structured telephone-screening interview was added prior to the face-to-face assessment to screen out participants obviously meeting exclusion criteria (e.g. currently receiving psychological therapy, see Blackwell et al., 2015). Inclusion criteria were: willing and able to give consent to the study, male or female aged between 18–65; fluent in written and spoken English; access to the internet in order to complete the online program (for the trial intervention); able to travel to the research center for assessment appointments; and meeting Diagnostic and Statistical Manual of Mental Disorders (4th ed., text rev.; American Psychiatric Association, 2000) criteria for a current major depressive episode assessed via semi-structured clinical interview (the Structured Clinical Interview for DSM–IV–TR Axis I Disorders, SCID; First et al., 2002). Exclusion criteria were: current psychological therapy; participation in concurrent treatment trials; current psychotic or substance-abuse disorder; history of mania/hypomania; or dosage change of antidepressant medication during the past month. Ethical approval was provided by the NRES Committee South Central - Oxford C (11/SC/0278).

Participants ranged in age from 18 to 65 years (*M*=35.49, *SD*=14.05), with 94.7% reporting their ethnicity as “White”, and 56.7% in paid employment, 28.0% student, 9.3% unemployed, 3.3% full-time homemaker or carer, and 2.7% retired.

### 2.2 Measures

#### 2.2.1 Prospective mental imagery vividness

Was assessed with the Prospective Imagery Task (PIT; Holmes et al., 2008; Stöber, 2000), a brief paper-based self-report measure. Participants read descriptions of 10 positive (e.g. *“People you meet will like you”*) and 10 negative (*“You will be the victim of crime”*) hypothetical future events and were asked to imagine each as if happening to them in the near future. Participants rated the subjective vividness of each of their images on a five-point scale, ranging from 1 (*no image at all*) to 5 (*very vivid*). Main predictions concern vividness ratings for the positive items, and negative items were included to control for general ability to generate future imagery (Blackwell et al., 2013). The mean of the vividness ratings for the relevant items is used as the ‘score’ for each subscale (positive or negative), and thus scores can range from 1 to 5, with a higher score indicating more vivid imagery.

The PIT has good internal consistency (0.83<α<0.90; Blackwell et al., 2013; see also Stöber, 2000). In our depressed sample, internal consistency was good for both positive (*α*=0.85) and negative (*α*=0.85) vividness scales.

#### 2.2.2 Optimism

The Life Orientation Test-Revisited (LOT-R; Scheier et al., 1994) is the most widely used measure of trait optimism. It includes three positively-framed statements (e.g. *‘‘In uncertain times, I usually expect the best”*) and three reverse-scored negatively-framed statements (e.g. *‘‘If something can go wrong for me, it will”*) rated on a 5-point scale from 0 (*strongly disagree*) to 4 (*strongly agree*). An additional four items were filler and not used in scoring. Responses to individual items are summed to derive a total score (range 0–24), with higher scores indicating higher trait optimism. The LOT-R has acceptable levels of test re-test reliability 0.68–0.79), internal consistency (0.70<α<0.80; Scheier et al., 1994), and convergent validity to measures of depression, life satisfaction and health status (Glaesmer et al., 2012).

#### 2.2.3 Control measures

Symptoms of depression were measured using the Beck Depression Inventory –Second Edition (BDI-II; Beck et al., 1996), a 21-item self-report scale that enquires about symptoms of depression over the previous two weeks. Scores can range from 0 to 63, with higher scores indicating more severe depression, and are classified as follows: 0–13: Minimal depression; 14–19: Mild depression; 20–28: Moderate depression; 29–63: Severe depression (Beck et al., 1996).

Trait anxiety was measured using the Trait scale of the State-Trait Anxiety Inventory (STAI-T; Spielberger et al., 1983), a 20-item scale on which participants are asked to rate various anxiety-related statement according to how they ‘generally feel’. Higher scores (possible range: 20–80) indicate higher levels of trait anxiety.

General everyday mental imagery use was measured using the Spontaneous Use of Imagery Scale (SUIS; Reisberg et al., 2003), a widely used 12- item scale measuring the tendency to use imagery in daily tasks and situations (e.g. “When I think about a series of errands I must do, I visualise the shops I will visit”). Responses are made on a 4-point scale, anchored from 1 (*never appropriate*) to 4 (*always appropriate*). Higher scores (range: 12–48) indicate a greater tendency to use imagery in everyday life.

Negative interpretation bias was measured using the Scrambled Sentences Test (SST; Rude et al., 2002). Participants unscrambled a list of 20 scrambled sentences (e.g. winner born I am loser a) under a cognitive load (remembering a 6 digit number). This measured the tendency of participants to interpret ambiguous information either positively (I am a born winner) or negatively (I am a born loser). A ‘negativity’ score is generated by calculating the proportion of sentences completed correctly with a negative emotional valence. Scores can thus range continuously between 0 and 1, with higher scores indicating a more negative bias.

General health status was measured using the EuroQol-5D-3 L visual analogue scale (EQ5D; Kind, 1996), a self-report measure of health-related quality of life. Participants self-rate their current health status on a Visual Analogue Scale (VAS), the endpoints of which are labelled "Best imaginable health state" (100) and "Worst imaginable health state" (0). Scores can therefore range from 0 to 100, with higher scores indicating better self-rated health.

### 2.3 Procedure

Eligibility was confirmed via a face-to-face assessment including the SCID interview. The baseline data reported in this paper were collected during a second face-to-face assessment. Baseline measures (with the exception of the scrambled sentences test) were administered prior to randomization into the trial, and thus at this time point both participant and researcher were blind to participant allocation. The LOT-R was completed again online (or pen-and-paper questionnaires returned via post) six months after the end of the four-week trial intervention, i.e. seven months after the baseline assessment.

The trial within which this data was collected compared an intervention involving repeated generation of positive mental imagery to a non-imagery “sham training” control condition, both delivered via the internet. See Blackwell et al. (2015) for full details of the clinical trial procedures and main outcomes.

### 2.4 Data analysis

Analyses were conducted via a series of linear regressions. The approach taken was to begin with only the primary variables of interest in the regression (e.g. for the cross-sectional analyses, vividness ratings for positive items on the PIT at baseline as the independent variable, and LOT-R scores at baseline as the dependent variable), with additional potential confounding factors added as independent variables in a series of additional steps to examine how the original relationship of interest was affected by inclusion of these variables at each stage. Details of the regressions and the variables included at each step are presented in the results section.

All variables included were continuous, with the exception of the following socio-demographic variables, which for replication purposes were coded as binary categorical variables to correspond as closely as possible to the categories used by Blackwell et al. (2013): gender (0=male, 1=female); educational level (0=≤13 years of education; 1=>13 years of education), relationship status (0=single/divorced/separated; 1=married/cohabiting), and nationality (0=non-UK; 1=UK).

The robustness of the analyses with regards to multivariate outliers and collinearity was verified via examination of residual plots, Cook's D values, and Tolerance/Variance Inflation Factors (Clark-Carter, 2010, Fox, 2008), in consultation with a statistician.

## 3 Results

Table 1 presents descriptive statistics for the sample, including socio-demographic information, and zero-order correlations with baseline optimism (LOT-R; *M*=8.07; *SD*=4.35).

### 3.1 Baseline optimism and vividness of prospective mental imagery

To test our hypothesis that greater ability to vividly imagine positive future events would be associated with higher levels of optimism, including when controlling for additional potentially confounding variables, a four-step linear regression was used. Baseline LOT-R score was the dependent variable, and baseline vividness for positive items on the PIT was the main independent variable of interest.

The first three steps tested whether the findings of Blackwell et al. (2013) would replicate in a depressed sample, by controlling for the same variables as in this previous study. In the first step, vividness ratings for positive items on the PIT were included as the only independent variable. In the second step, socio-demographic factors as used by Blackwell et al. (2013) were included as additional potentially confounding independent variables. In the third step, vividness ratings for negative items on the PIT and scores on the SUIS were included as additional independent variables to control for vividness of negative prospective imagery and general imagery use. A fourth step provided an extension of the previous research, by additionally controlling for depression, anxiety and interpretation bias (not included in the study by Blackwell et al., 2013). Including these additional variables allowed us to test further the specificity of the relationship between positive imagery vividness and optimism.

To summarize, variables entered were: Step 1, vividness ratings for PIT positive items; Step 2, socio-demographic variables (age, gender, marital status, self-rated health, nationality, education); Step 3, SUIS score and PIT negative item vividness ratings; Step 4, BDI-II, STAI-T and SST scores. All 150 participants were included in the analysis.

Results are summarized in Table 2. In the initial model (Step 1), with vividness ratings for PIT positive items as the only independent variable, higher vividness ratings were associated with higher LOT-R scores (*β*=0.356 [95% CI: 0.204, 0.508], *p*<0.001). The relationship between vividness ratings for PIT positive items and LOT-R scores remained significant when socio-demographic variables were entered in Step 2 (*β*=0.378 [0.232, 0.524], *p*<0.001), and when vividness ratings for PIT negative items and SUIS scores were entered in Step 3 (*β*=0.554 [0.391, 0.717], *p*<0.001). In the final model, higher vividness ratings for PIT positive items were significantly associated with higher LOT-R scores (*β*=0.314 [0.151, 0.476], *p*<0.001). The only other statistically significant independent variable was trait anxiety (STAI-T), which was negatively associated with LOT-R score (*β*=−0.315 [−0.483, −0.145], *p*<0.001).

### 3.2 Predicting optimism at seven months

A three-step linear regression model was used to test whether vividness of positive prospective imagery at baseline predicted optimism seven months later, over and above baseline optimism and other potentially relevant variables. This analysis used participants from the control condition (as this involved no positive imagery practice),^1^ who completed the LOT-R at the final study assessment (*n*=63). There were no significant differences at baseline in any variables included in the previous regression between those who did versus did not complete (*n*=11) the final LOT-R, except for self-rated health (EQ-5D): those who did not complete the LOT-R at seven months scored lower on this measure at baseline (*M*=48.55, *SD*=15.02) than those who did complete it (*M*=60.68, *SD*=19.04), *t* (72)=2.00, *p=*0.049.

The dependent variable in the regression was LOT-R score at seven months, and the main independent variable of interest was vividness for positive items on the PIT at baseline. Potentially relevant confounding variables included in the regression were LOT-R and BDI-II scores at baseline, and variables with a significant relationship with optimism in our previous regression (i.e. STAI-T at baseline).

Results are summarized in Table 3. In Step 1 of the model, baseline vividness ratings for positive items on the PIT were entered, showing that higher vividness ratings at baseline predicted higher LOT-R scores seven months later (*β*=0.442 [95% CI: 0.212, 0.671], *p*<0.001). In Step 2, baseline LOT-R scores were entered. When adjusted for baseline optimism, higher vividness ratings for positive PIT items (*β*=0.260 [0.061, 0.459], *p*=0.011) still predicted higher LOT-R scores seven months later. In Step 3, baseline STAI-T and BDI-II scores were entered. When additionally adjusted for baseline anxiety and depression, higher vividness ratings for positive items of the PIT (*β*=0.249 [0.054, 0.444], *p*=0.013) still predicted higher LOT-R scores seven months later. In this final model, only higher LOT-R scores (*β*=0.501 [0.230, 0.772], *p*<0.001) at baseline also significantly predicted higher LOT-R scores seven months later.

To test the *temporal* nature of the relationship (i.e. that positive prospective imagery vividness predicted future optimism, but not vice versa), the opposite direction of prediction was tested (cf. Nelis et al., 2015). We carried out an equivalent regression, but with PIT positive imagery vividness ratings at seven months as dependent variable. In a first step, including only baseline LOT-R score as a predictor, baseline LOT-R score (*β*=0.312 [95% CI: 0.067, 0.557], *p*=0.014) significantly predicted PIT positive imagery scores seven months later. However, when adjusting for baseline PIT positive imagery scores in a second step, baseline LOT-R score (*β*=0.148 [−0.078, 0.373], *p*=0.195) no longer predicted PIT positive imagery scores seven months later. In the third and final step, in which baseline BDI-II and STAI-T scores were additionally entered as predictors, baseline LOT-R score (*β*=−0.022 [−0.328, 0.284], *p*=0.886) did not predict positive imagery vividness seven months later.

### 3.3 Specificity of longitudinal prediction

In additional exploratory analyses, we investigated whether positive imagery vividness at baseline might be a broader predictor of positive outcomes at seven months, rather than specifically predicting optimism only. We therefore repeated the regression presented in Table 3, but first with BDI-II at seven months as the dependent variable, and adding baseline PIT positive imagery vividness, baseline BDI-II, and baseline LOT-R and STAI-T as predictors in three successive steps. In isolation (Step 1), higher baseline positive imagery vividness did predict lower BDI-II scores at seven months (*β*=−0.261 [95% CI: −0.506, −0.016], *p*=0.037), but when adjusted for baseline BDI-II (Step 2), this prediction was no longer statistically significant (*β*=−0.186 [−0.414, 0.043], *p*=0.109). When additionally adjusted for baseline LOT-R and STAI-T scores (Step 3), baseline PIT positive vividness again did not significantly predict BDI-II scores seven months later (*β*=−0.218 [−0.450, 0.014], *p*=0.066).

We then conducted an equivalent regression with STAI-T scores at seven months as the outcome variable, and with baseline PIT-PV, baseline STAI-T, and baseline LOT-R and BDI-II added as predictors in successive steps. In isolation (Step 1), higher baseline positive imagery vividness did predict lower STAI-T scores at seven months (*β*=−0.323 [−0.567, −0.078], *p*=0.011), but when adjusted for baseline STAI-T (Step 2), this prediction was no longer statistically significant (*β*=−0.209 [−0.433, 0.015], *p*=0.067). When additionally adjusted for baseline LOT-R and BDI-II scores (Step 3), baseline PIT positive vividness did significantly predict STAI-T scores seven months later (*β*=−0.240 [−0.470, −0.011], *p*=0.041).

### 3.4 Optimism/pessimism dimensions of the LOT-R

Following suggestions (e.g. Kubzansky et al., 2004) that the LOT-R can be seen as assessing two related but separate constructs, optimism (via the positively-worded items) and pessimism (via the negatively-worded items), in exploratory analyses we repeated the cross-sectional and main longitudinal regression using these separate subscales as dependent variables rather than the whole LOT-R score. The main results remained unchanged. For the cross-section analysis, higher vividness ratings of PIT positive items were significantly associated with higher LOT-R optimism subscale scores (*β=*0.307 [95% CIs: 0.136, 0.478], *p*=0.001) and lower LOT-R pessimism subscale scores (*β=*−0.264 [−0.440, −0.087], *p*=0.004). For the longitudinal analysis, higher vividness ratings of PIT positive items at baseline predicted higher LOT-R optimism subscale scores at 7 months (*β=*0.238 [0.065, 0.412], *p*=0.008) and lower LOT-R pessimism subscale scores at 7 months (*β=*−0.232 [−0.453, −0.010], *p*=0.041).

## 4 Discussion

Scores on a simple laboratory-administered measure assessing how vividly individuals with depression could imagine positive future life events were statistically significantly related not only to current levels of dispositional optimism, but also to how optimistic people were seven months later. These findings confirm a test of replication of Blackwell et al. (2013), and critically, extend them as follows: First, the cross-sectional relationship between positive prospective imagery vividness and dispositional optimism, as measured by the Life Orientation Test-Revised, generalized from a community sample to a clinical sample of people with depression. Second, results provide further evidence for potential specificity of the relationship by controlling for depression, anxiety, and interpretation bias. Third, the temporal component shows that positive prospective imagery vividness predicted future optimism (but not vice versa). Together, results highlight positive prospective imagery vividness as a potential cognitive mechanism underlying optimism.

Why might the ability to vividly conjure up scenes of positive future events in our lives be important in shaping how we feel about our future? Functional theories of prospection view mental imagery as a core component of the ‘prospective brain’, enabling individuals to predict and plan for the future using perceptual simulations (Moulton and Kosslyn, 2009, Suddendorf and Corballis, 2007). Importantly, perceptual simulations of experiences using mental imagery can evoke neurophysiological and emotional responses as if the experience was real (Ji et al., 2016). Individuals may use the experience of simulating specific events as information to help evaluate the future. For example, they may interpret the ease with which a particular hypothetical event comes to mind as reflective of the likelihood of similar events occurring in real life (e.g. Kahneman and Tversky, 1982; Szpunar and Schacter, 2013). In other words, people form expectancies of the future by “seeing” it through the “mind's binoculars”. The powerful emotional impact of mental imagery (Holmes and Mathews, 2005) and its ‘realness’ (Mathews et al., 2013) underscore how imagery-based future simulations may influence how optimistic or otherwise we feel.

While our hypotheses concerned the relationship between positive imagery vividness and optimism, the relationship between vividness ratings for negative items on the Prospective Imagery Test (PIT) and LOT-R scores observed in the cross-sectional analysis is also of note. We found that there was no statistically significant zero-order relationship between negative prospective imagery and optimism, but when controlling for positive imagery vividness in the regression, a significant (negative) relationship between *negative* imagery vividness and optimism emerged. Interestingly, this replicates the pattern of results found by Blackwell et al. (2013) in relation to negative imagery vividness. In the current study, the addition of depression, trait anxiety, and interpretation bias in the fourth step of the regression reduced the relationship between negative imagery vividness and optimism to statistical non-significance. Exploration of this reduction to non-significance indicated that it occurred due to the addition of the STAI-T (trait anxiety) to the regression. Thus, it appears that the significant relationship between negative imagery vividness and optimism that emerges when positive imagery vividness is controlled for reflects an association between negative imagery vividness and anxiety, rather than being specific to optimism. This is consistent with theoretical accounts (cf. Miloyan et al., 2013), and with the finding from previous research that people with anxiety disorders reported significantly higher vividness ratings on for negative items on the PIT than people with major depression and healthy controls (Morina et al., 2011). Conversely, the relationship between positive imagery vividness and optimism appears to be more specific.

Our finding that vividness of positive prospective imagery predicted optimism seven months later can be interpreted as indicating that reduced ability to generate vivid positive prospective imagery is a “risk factor” (Kraemer et al., 1997, Offord and Kraemer, 2000) for reduced recovery of optimism over time. This could mean either that reduced positive prospective imagery vividness is a marker of individuals who may particularly benefit from an intervention to increase optimism or compensate for negative effects of reduced optimism (if it is a “fixed” or “variable” risk factor; Offord and Kraemer, 2000), or that increasing individuals’ ability to vividly imagine positive future events may provide a route to increase their dispositional optimism (if a “causal” risk factor). From another perspective, the ability to vividly imagine positive future events could be said to be a “protective factor” (Offord and Kraemer, 2000) for optimism. While psychopathology research has tended to focus on dysfunctional processes, positive protective factors such as optimism may be particularly important in recovery and remission (e.g. Boelen, 2015; Teismann et al., 2016; Trumpf et al., 2009).

In addition to testing whether positive imagery vividness at baseline predicted optimism at seven months, in exploratory analyses we investigated the specificity of this prediction. That is, we carried out additional regressions to test whether positive imagery vividness at baseline also predicted depression or trait anxiety seven months later. These results were more equivocal, and we note that if we were to correct for the multiple analyses (i.e. Bonferroni correction for the three prediction regressions), only prediction of the LOT-R by baseline PIT positive imagery vividness would survive this more stringent probability threshold (*p*<0.017). However, the regression coefficients for PIT positive imagery vividness scores were similar in all three regressions, and overall the results do not provide strong evidence for the suggestion that positive prospective imagery vividness predicts *only* future optimism. In fact, such as result would be surprising given the relationship between optimism, depression, and anxiety. In future studies it would be interesting to investigate the mechanisms linking these facets of psychopathology and wellbeing, and the underlying processes, ideally within the context of an experimental manipulation to test causality and allow conclusions about the direction of effects.

Could increasing the vividness with which individuals can imagine positive events in their future lead to increases in their optimism? This possibility has yet to be demonstrated. In the trial from which the current data was derived, there was no difference between the two intervention conditions in the change in positive imagery vividness (as measured by the PIT) over time (Blackwell et al., 2015), and thus potential causal relations could not be examined. Conversely, in a sample of healthy older adults, Murphy et al. (2015) found that while completing a four-week positive imagery intervention based on that of Blackwell et al. (2015) led to increases in vividness ratings for positive items on the PIT, it did not lead to increases in optimism as measured by the LOT-R, compared to a control condition. This dissociation could potentially indicate that positive imagery vividness is not a direct causal driver of dispositional optimism. However, participants in Murphy et al. (2015) had high levels of optimism at baseline, therefore the absence of further increases in optimism may reflect a ceiling effect. Additionally, it may be that a sustained increase in positive imagery vividness and a longer time-period of measurement would be required to observe changes in self-reported trait optimism as measured by the LOT-R. Increases in self-reported optimism in healthy populations have been induced via brief (Peters et al., 2010) and longer (e.g. two weeks; Meevissen et al., 2011) positive imagery exercises involving imagining a ‘best possible self’ in the future (see also Malouff and Schutte, 2016). However, the mechanisms or longevity of such effects are not yet known. It is worth noting that high vividness ratings on the PIT may reflect frequent engagement in similar prospective imagery in everyday life, increasing its accessibility or increasing its vividness via rehearsal (cf. Szpunar and Schacter, 2013), and it could be this frequent engagement in positive imagery (rather than vividness per se) that relates to optimism. Thus, the nature of the relationship between positive mental imagery and optimism, and whether training positive mental imagery can lead to sustained beneficial changes in optimism in clinical populations such as depressed individuals remains to be determined (Holmes et al., 2016).

In the current study we measured optimism using the widely-used Life Orientation Test – Revised (LOT-R; Scheier et al., 1994). While there are a number of ways to operationalize or measure the construct of optimism, self-report dispositional optimism as measured by the LOT-R appears a robust predictor of psychological and physical wellbeing (Carver et al., 2010), over and above alternative factors proposed to explain optimism's beneficial effects, such as neuroticism and self-efficacy (Scheier et al., 1994). Whether these benefits are in fact a consequence of having an optimistic outlook (e.g. one's positive expectancies about the future leading to a more adaptive course of action or buffering against stress), or rather result from one or more of the processes that potentially underpin optimism, such as biases in attention, memory, and interpretation, (Fox, 2012) or belief-updating mechanisms (Sharot et al., 2011), remains to be determined. Future studies aiming to manipulate processes hypothesized to contribute to optimism and explore the potential benefits may therefore profit from measuring the direct effects of the manipulation on optimism-related behavioural or emotional outcomes of interest, particularly as dispositional (i.e. trait) optimism (as measured by the LOT-R) may be slow to change. The trial data included a measure of negative interpretation bias, allowing us to control for this aspect of depression-relevant cognition when examining the relationship between positive prospective imagery vividness and optimism. However, given that depression is characterized by a number of other cognitive biases and deficits (Browning et al., 2013, Gotlib and Joormann, 2010), including in relation to future-oriented thinking (e.g. MacLeod and Salaminiou, 2001; Miranda and Mennin, 2007), exploring potential links between imagery vividness, optimism, and other cognitive biases and dysfunctions in depression will help to further elucidate mechanisms and guide treatment development.

A limitation of the study is that longitudinal data cannot themselves provide evidence of causal relationships. Further, participants received an intervention (albeit in a control condition) in the first four weeks of the seven-month time period, and thus similar results may not be found in naturalistic settings with no intervention. Participants were recruited for a research study of an internet-delivered intervention for depression, and thus the sample may not be representative of the broader general population. Finally, we assume that increases in optimism would be desirable; while there is some debate about whether the apparent “optimism bias” (Sharot, 2011) observed in the general population is in fact “unrealistic” or otherwise (Harris and Hahn, 2011), excessive optimism may not always be optimal (Carver and Scheier, 2014). The results of this study need to be interpreted in the context of the trial from which the data originates; that is, they derive from a secondary analysis of data from a trial that was not specifically designed to test this paper's hypotheses. However, the opportunity to examine both cross-sectional and longitudinal data within a sample of 150 depressed individuals presents a valuable opportunity for clinical research.

In summary, the present results suggest that even when individuals are depressed, those able to envision a brighter future are relatively more optimistic, and regain optimism more quickly, than those less able to do so. The ability to vividly imagine positive events in the future, and thus see it through “rose-tinted binoculars”, provides a potentially modifiable cognitive target warranting further research to inform mental health treatment innovation.

## Figures and Tables

**Table 1:** Descriptive statistics for baseline variables included in cross-sectional analyses, and zero-order correlations with baseline score on the Life Orientation Test – Revised.

Variable	*M* (SD) or *N* (%)	*r*_0_
*Optimism and positive imagery vividness*		
LOT-R	8.07 (4.35)	–
PIT Positive Imagery Vividness	2.85 (0.84)	0.36^***^

*Socio-demographic variables*		
Age (years)	35.49 (14.05)	0.09
Gender - Female	103 (68.67%)	−0.11
>13 years education	121 (80.67%)	0.13
Married/Cohabiting	56 (37.33%)	−0.00
Physical Health (EQ−5D VAS)	60.15 (19.53)	0.30^***^
UK nationality	126 (84.00%)	−0.08

*General imagery use and negative imagery vividness*		
SUIS score	39.41 (8.99)	0.08
PIT Negative Imagery Vividness	3.34 (0.86)	−0.15

*Interpretation bias, Depression, and Anxiety*		
BDI-II score	30.54 (9.41)	−0.52^***^
STAI-T score	61.29 (6.59)	−0.60^***^
SST Negativity score	0.59 (0.24)	−0.56^***^

*N=*150. *r*_0_=zero order correlations with baseline LOT-R score; LOT-R=Life Orientation Test - Revised; PIT Negative/Positive Imagery Vividness=imagery vividness ratings for negative/positive items on the Prospective Imagery Test (PIT); EQ-5D=Euroqol-5D-3L (self-rated health rated from “Worst imaginable health state”, scored as 0, to “Best imaginable health state”, scored as 100); SUIS=Spontaneous Use of Imagery Scale; BDI-II=Beck Depression Inventory - II; STAI-T=State-Trait Anxiety Inventory - Trait version; SST Negativity=Scrambled Sentences Task negativity score. ^*^p<0.05; ^**^p<0.01; ^***^p<0.001.

**Table 2:** Positive prospective mental imagery vividness and optimism at baseline: cross-sectional regression analysis with Life Orientation Test – Revised score as dependent variable.

Independent variable	Model 1 *β*	Model 2 *β*	Model 3 *β*	Model 4 *β*
PIT Positive Vividness	0.36^***^	0.38^***^	0.55^***^	0.31^***^
Age (years)		0.28^***^	0.20^**^	0.08
Gender (female)		−0.14	−0.11	−0.08
>13 years education		0.12	0.10	0.11
Married/Cohabiting		−0.06	−0.04	−0.02
EQ−5D		0.31^***^	0.23^**^	0.10
UK nationality		−0.04	−0.03	0.00
SUIS			0.02	0.02
PIT Negative Vividness			−0.36^***^	−0.12
BDI-II				−0.15
STAI-T				−0.31^***^
SST Negativity				−0.13
Adjusted *R*^2^	0.12	0.26	0.33	0.49
∆*R*^2^	0.13	0.16	0.08	0.16
*F* for ∆*R*^2^	21.49^***^	5.45^***^	8.88^***^	15.82^***^
*F* for model	21.49^***^	8.30^***^	9.14^***^	12.99^***^

*N*=150. Four-step linear regression with LOT-R score as dependent variable: Model 1 includes positive prospective imagery vividness only. Model 2 additionally includes socio-demographic variables. Model 3 additionally includes control imagery variables. Model 4 additionally includes depression, trait anxiety, and negative interpretation bias. LOT-R=Life Orientation Rest - Revised; PIT Negative/Positive Vividness=imagery vividness ratings for negative/positive items on the Prospective Imagery Test (PIT); EQ-5D=Euroqol-5D-3L (self-rated health rated from “Worst imaginable health state”, scored as 0, to “Best imaginable health state”, scored as 100); SUIS=Spontaneous Use of Imagery Scale; BDI-II=Beck Depression Inventory - II; STAI-T=State-Trait Anxiety Inventory - Trait version; SST Negativity=Scrambled Sentences Task negativity score. ^*^p<0.05; ^**^p<0.01; ^***^p<0.001.

**Table 3:** Positive prospective mental imagery vividness and optimism seven months later: longitudinal analysis.

Predictor	M (SD)	*r*_0_	Model 1 β	Model 2 β	Model 3 β
PIT Positive Vividness	2.88 (0.83)	0.44^***^	0.44^***^	0.26^*^	0.25^*^
LOT-R Baseline	7.90 (4.70)	0.64^***^		0.56^***^	0.50^***^
STAI-T	61.51 (7.11)	−0.54^***^			−0.29
BDI-II	30.86 (10.38)	−0.32^*^			0.23
Adjusted *R*^2^			0.18	0.45	0.48
Δ*R*^2^			0.20	0.28	0.04
*F* for Δ*R*^2^			14.78^***^	31.29^***^	2.27
*F* for model			14.78^***^	26.71^***^	15.06^***^

*N=63*. Three-step linear regression model with LOT-R score at the end of the study (seven months post-baseline) as dependent variable. Model 1 includes positive prospective imagery vividness only. Model 2 additionally includes baseline LOT-R score. Model 3 additionally includes baseline variables previously shown to have a significant relationship with optimism at baseline in the previous cross-sectional analysis (STAI-T) and baseline levels of depression (BDI-II). r_0_=zero order correlations with LOT-R score at seven months post-baseline. PIT Positive Vividness=imagery vividness ratings for positive items on the Prospective Imagery Test; LOT-R=Life Orientation Test – Revised; STAI-T – State-Trait Anxiety Inventory – Trait version; BDI-II=Beck Depression Inventory – II. *p*<0.05; ^**^p<0.01; ^***^p<0.001.

## Footnotes

1 We additionally carried out the same regression with all participants providing data for the LOT-R at the final study assessment (*n*=130), controlling for condition (i.e. which intervention the participant was randomized to) by including condition as a binary predictor variable (i.e. positive imagery or control) in the regression. This yielded an equivalent result, with baseline PIT positive imagery vividness significantly predicting LOT-R seven months later (*β*=0.211 [0.063, 0.358], *p*=0.005). There were no differences between conditions in scores on the LOT-R at any time point (all *t*s<1), consistent with the main trial analyses showing no difference between conditions in reduction in symptoms of depression (Blackwell et al., 2015). However, in our main prediction analyses we restricted the regression to participants in the control condition, as the positive imagery intervention involved repeated generation of positive mental imagery over a four-week period, and this might complicate interpretation of effects of baseline imagery vividness on subsequent outcomes. Further, as there were some between-group differences in outcomes in post-hoc analyses (e.g. for anhedonic symptoms), we would be cautious about interpreting the positive imagery intervention as equivalent to the control condition.
